# Potential Evaluation for Preparing Geopolymers from Quartz by Low-Alkali Activation

**DOI:** 10.3390/ma16041552

**Published:** 2023-02-13

**Authors:** Wei Ge, Jun Chen, Fanfei Min, Shaoxian Song, Hui Liu

**Affiliations:** 1 State Key Laboratory of Silicate Materials for Architectures, Wuhan University of Technology, Wenzhi Street 34, Wuhan 430070, China; 2 Department of Materials Science and Engineering, Anhui University of Science and Technology, Taifeng Street 168, Huainan 232001, China; 3 Department of Civil Engineering, Changzhou University, Changzhou 213164, China

**Keywords:** low-alkali activation, granite sawdust, alkali fusion, quartz, geopolymer

## Abstract

Alkali fusion of granite sawdust at a high alkali dosage can significantly improve geopolymerization activity, but also result in a high alkali consumption and a poor geopolymer performance. In this work, quartz, the most inert component in granite sawdust, was selected to explore the effect of low-alkali activation on its reactivity and the compressive strength of geopolymer. It was found that the amount of activated quartz is mainly determined by the amount of alkali used for activation. The surface of a quartz particle can be effectively activated by an alkali fusion process at a low alkali dosage of 5%. The metakaolin-based geopolymer synthesized with quartz activated by an alkali dosage of 5% shows a high compressive strength of 41 MPa, which can be attributed to the enhanced interfacial interaction between quartz and the geopolymer gel, suggesting that low-alkali activation is a potential way to improve the geopolymerization ability of granite sawdust.

## 1. Introduction

Granite is a hard and compact massive silicate rock with quartz and feldspar minerals as the primary constituents [1]. In recent decades, with the rapid urbanization process in China, a large amount of granite stones have been sawn into bricks, plates or polished into ornaments for civil engineering. Granite sawdust mainly composed of quartz and feldspar fines is a by-product produced in the sawing and polishing process. At present, due to the lack of effective utilization methods, billions of tons of granite sawdust have been accumulated, which have occupied a lot of farmland and caused serious pollution to the land, local water resources and air [2].

Geopolymer is a kind of amorphous or semi-crystalline aluminosilicate material [3,4,5], and consists of tetrahedral AlO_4_ and SiO_4_ units polycondensed into three-dimensional micro-structures with a charge stabilized by alkaline earth ions [6,7]. It can be synthesized through the alkali activation of aluminosilicate minerals and industrial by-products. In recent decades, the raw materials applied to prepare geopolymers have been extended from kaolinite minerals to many other industrial wastes containing aluminosilicate such as fly ash, red mud, blast furnace slag and mine tailing [8,9,10,11]. Geopolymer materials have been studied extensively and considered as an alternative to cement, due to several merits such as high and early compressive strength, long-term durability, optimal acid resistance, elevated temperature resistance and low cost [12,13]. Moreover, compared to Portland cement, the production of geopolymers produces 80% less CO_2_ [14,15,16].

Because of the huge consumption of building materials, preparing geopolymers with granite sawdust is a potential strategy to solve the problems of land occupation and environmental pollution [17,18]. However, quartz, an inert silicate mineral in granite sawdust, cannot participate in geopolymerization, even weakening the structure of the geopolymer gel, resulting in a low compressive strength [19,20]. Alkali fusion is an effective method to improve the reactivity of quartz [21,22]. Tchadjie [23] has activated granite sawdust through calcining with Na_2_O in the range of 10–60% at 550 °C. However, the compressive strength of the synthesized granite-sawdust-based geopolymer was only 10.94 MPa when the amount of Na_2_O used for activation was 10%. Additionally, when the alkali dosage for activation is higher than 20%, the water resistance of the synthesized geopolymer becomes worse. The geopolymer synthesized with quartz activated by 20% NaOH shows a compressive strength of 29 MPa after 7 days of curing in ambient conditions [24]. The purpose of alkali fusion is to increase the quartz reactivity as high as possible by destroying its crystal structure, which results in a high alkali consumption and efflorescence [25,26,27].

Wan et al. [28] activated the quartz surface using planetary ball milling at 1000 r/min for 1 h. After activation, nearly 5% of the active Si component was leached from the quartz, and the compressive strength of the geopolymer prepared with activated quartz increased significantly from 31.5 to 55.2 MPa. The enhanced mechanical performance is attributed to the active Si-containing materials produced by mechanical activation on the activated quartz surface promoting the formation of a Si-rich gel around the quartz. Although mechanical activation is a method with a high energy consumption and low efficiency, the aforementioned study demonstrated that only activating the quartz surface can also improve the mechanical performance of geopolymers [29]. Because feldspar can be easily activated by alkali fusion, if the surface of the quartz found in granite sawdust can effectively be activated by alkali fusion with a low alkali consumption, the properties of geopolymers synthesized with granite sawdust can also be improved.

Thus, in this study, the activation of quartz by the alkali fusion method with a low alkali consumption and its effect on the compressive strength of geopolymers were investigated. It provides a new perspective on the synthesis of geopolymers with low-alkali-activated material and a further understanding on the connection between the surface activation of inert minerals and mechanical performance.

## 2. Materials and Methods

### 2.1. Materials

**Figure 1:**
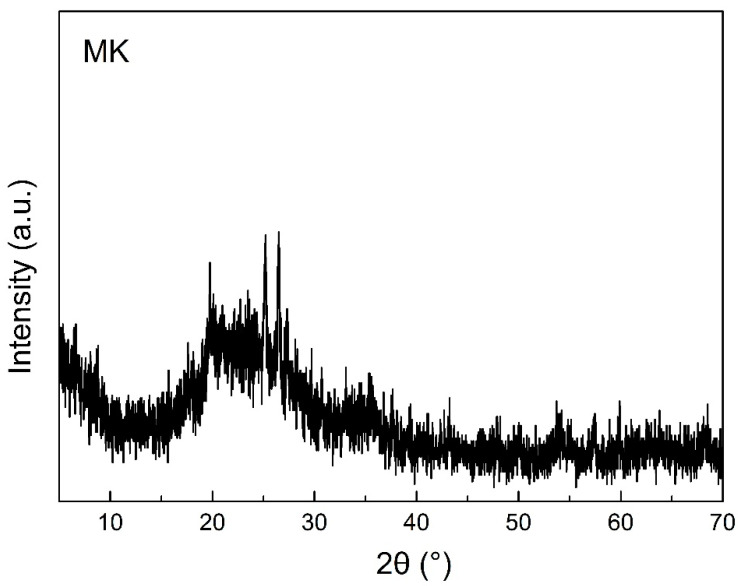
XRD pattern of MK.

**Figure 2:**
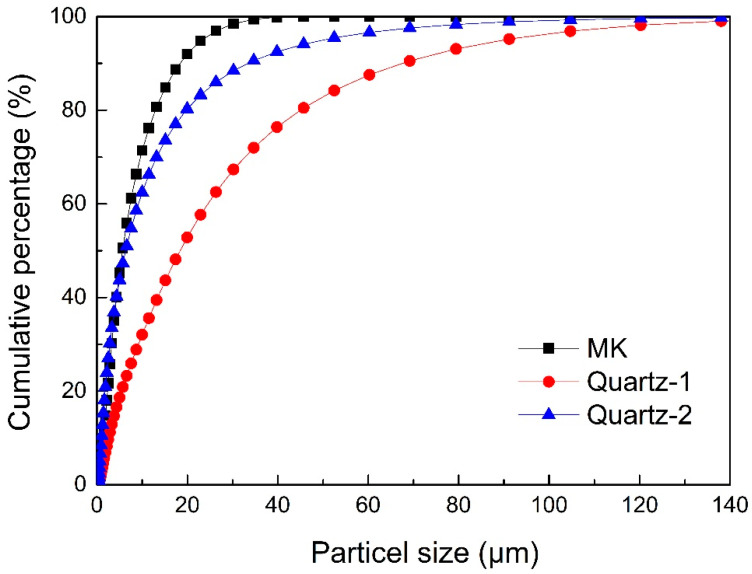
Particle size distributions of MK, quartz-1 and quartz-2.

Kaolinite from Gongyi, Henan, China, was calcined at 800 °C for 2.5 h to prepare metakaolin (MK). The chemical composition of MK analyzed via X-ray fluorescence (XRF) is listed in Table 1. The total content of SiO_2_ and Al_2_O_3_ was 92 wt%, and the mass ratio of SiO_2_ to Al_2_O_3_ was close to that of pure MK. The XRD pattern of MK (Figure 1) had a broad peak centered around 22° indicating the amorphous structure of MK [30]. The sharp peaks at 19.78°, 25.23° and 26.54° belonged to quartz [31]. The particle size distribution of MK is shown in Figure 2, and the d50 and d90 were 5.7 μm and 19.0 μm, respectively.

Quartz particles were collected from Luoyang, Henan, China. The original quartz particles were labeled as quartz-1. To obtain fine-grained quartz particles, the original quartz was milled, and the obtained sample was labeled as quartz-2. The size distributions of quartz are presented in Figure 2. The d50 and d90 of quartz-1 were 20 μm and 70 μm, respectively. The d50 and d90 of quartz-2 were 6.60 μm and 34.6 μm, respectively.

Sodium hydroxide (NaOH) of an analytical grade and purchased from Sinopharm Chemical was used to adjust the modulus of water glass. Water glass with a modulus of 2.31 and content of 42 wt% was bought from Wuxi Yatai United Chemical Co., Ltd. (Wuxi, China). Deionized water was used in all the experiments.

### 2.2. Alkali Fusion

Quartz particles and sodium hydroxide were evenly mixed using a vibration mill (WHROCK, RK/ZM-400, China) at 600 r/min for 10 s. Then, the obtained mixture was heated in a muffle furnace at 550 °C for 60 min within 15 min. The heating rate was 10 °C/min. After cooling to room temperature, the activated quartz was stored in a sealed bag to avoid atmospheric carbonation. To activate the quartz under different alkali dosages, the mass of NaOH used were 2.5%, 5%, 10%, 15% and 20% of quartz, respectively, and the corresponding activated materials of quartz-1 and quartz-2 were labeled as AQ1-2.5, AQ1-5, AQ1-10, AQ1-15, AQ1-20, AQ2-2.5, AQ2-5, AQ2-10, AQ2-15 and AQ2-20, respectively. As a control group, quartz-1 and quartz-2 were calcined at 550 °C for 60 min, and the resulting samples were labeled as calcined quartz-1 and calcined quartz-2, respectively.

The active silicon contents in quartz and activated quartz were applied to evaluate the activation effect, which was measured by the following process. A total of 5 g of quartz or activated quartz was added into 50 mL of a 5 mol/L NaOH solution. The obtained suspension was stirred with a magnetic stirrer for 6 h to dissolve the active Si. Then, 10 mL of the suspension was taken out with a syringe and filtered to obtain the leaching solution. The Si concentration in the solution was measured and then used to calculate the activated Si content, which was applied to represent the content of activated quartz.

### 2.3. Synthesis of Geopolymer

To prepare the MK-based geopolymers, 50 g of MK (0.225 mol) and 50 g of quartz or activated quartz were mixed and stirred using a cement mixer for 5 min, followed by the addition of water glass as an activator, with mechanical stirring for 5 min more [32]. The formed paste was poured into a steel mold with a cavity size of 2 cm × 2 cm × 2 cm. After grouting, the mold was vibrated 100 times to release the air in the paste. Then, the mold with paste inside was sealed in a polyethylene bag. The sealed mold was first cured at 60 °C for 6 h, and then maintained at room temperature for 14 days. In all syntheses, the mole ratio of Si/Al was 1.5:1, and the mole ratio of Na_2_O/H_2_O was 1:11. For quartz and activated quartz, only active Si was used for calculation, which was determined via the leaching test. The MK geopolymer was prepared without using quartz particles.

### 2.4. Material Characterization

The particle size distributions of MK and quartz were characterized using a Malvern Mastersizer 2000 accompanied by ultrasonic dispersion and mechanical stirring at 1800 r/min, (Malvern Instruments, Worcestershire, UK). The chemical composition of MK was analyzed via X-ray fluorescence (XRF, Zetium, PANalytical.B.V, The Netherlands) with rhodium as the X-ray source and an accuracy of 0.05%. X-ray diffraction (XRD, D8-Advance, Bruker, Germany) with Cu as an X-ray source and an angle measurement accuracy of less than 0.01°, was used to determine the material phase. The infrared spectra of activated quartz and geopolymer samples were obtained using a Fourier Transform Infrared Spectrometer (FTIR, Nexus JSM-5610, Thermo Nicolet, USA). The material surface morphologies were characterized using a Phenom Desktop Scanning Electron Microscopy (SEM, Phenom Prox, Phenom Scientific, USA) with an acceleration voltage at 15 kV and high beam currents. An Inductively Coupled Plasma-Optical Emission Spectrometer (ICP-OES, Prodigy 7, USA) with a precision of less than 2% was applied to measure the Si concentration in the solution. The compressive strength of the geopolymer sample was measured using a mechanical tester (YES-100, TENSON, Jinan, China).

## 3. Results and Discussion

### 3.1. Microanalysis of Activated Quartz

#### 3.1.1. XRD

**Figure 3:**
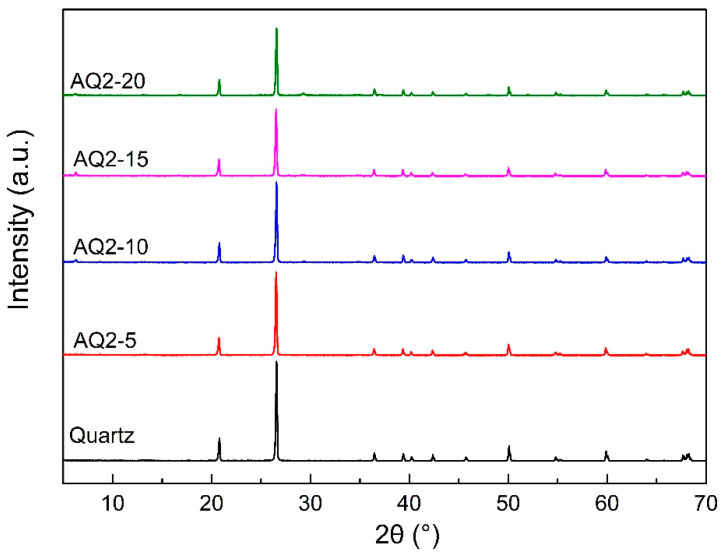
XRD patterns of quartz and quartz-2 activated with various contents of NaOH.

The XRD patterns of quartz and quartz activated with various amount of NaOH are shown in Figure 3. All the peaks on the pattern of quartz and activated quartz belong to quartz, indicating a high purity of the raw material [33]. As the alkali dosage used for activation increases, the peak intensities of quartz show a downward trend, suggesting the decrease in quartz content in the activated quartz. In addition to the increase in alkali dosage, the conversion of part of the quartz into amorphous or semi-crystalline materials under the activation treatment may also lead to the decrease in peak intensity. It can be observed that the XRD peaks of quartz before and after activation have no obvious change, which indicates that the material formed by the activation of quartz is amorphous or semi-crystalline.

#### 3.1.2. FTIR

**Figure 4:**
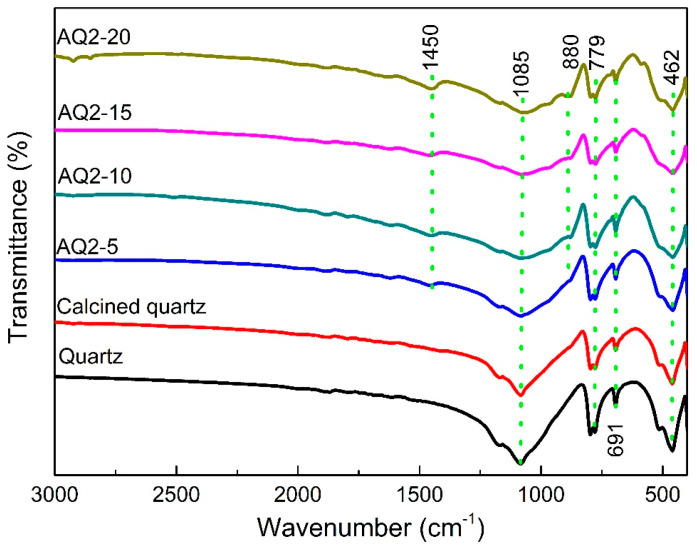
FTIR spectra of quartz, calcined quartz and quartz-2 activated with various content of NaOH.

The FTIR spectra of quartz, calcined quartz and activated quartz with various contents of NaOH are shown in Figure 4. The weak bands at 1450 cm^−1^ and 880 cm^−1^ observed on the pattern of activated quartz are assigned to the stretching vibrations of O-C-O in carbonates, indicating a slight carbonation of the activated quartz, which may be caused by the exposure of activated quartz to the air [34]. The absorption band at 462 cm^−1^ is assigned to the asymmetrical bending vibration of Si-O-Si, the absorption band at 691 cm^−1^ corresponds to the symmetrical bending vibration of Si-O, and the bands at 779 cm^−1^ and 1080 cm^−1^ belong to the symmetrical and asymmetrical stretching vibration of Si-O, respectively [35]. The band initially present at 1085 cm^−1^ on the spectrum of quartz and calcined quartz shifts toward lower wavenumbers, and becomes broad and less profound after alkali activation [36]. With the increase in alkali dosage used for activation, these changes become more prominent, which can be attributed to the structural reorganization and the increase of non-bridging oxygen [37]. Therefore, the FTIR results demonstrate that part of quartz was transformed into active substances under alkali fusion treatment, and the content of the active materials increased with the increase in alkali dosage, which is consistent with the XRD results.

#### 3.1.3. SEM

**Figure 5:**
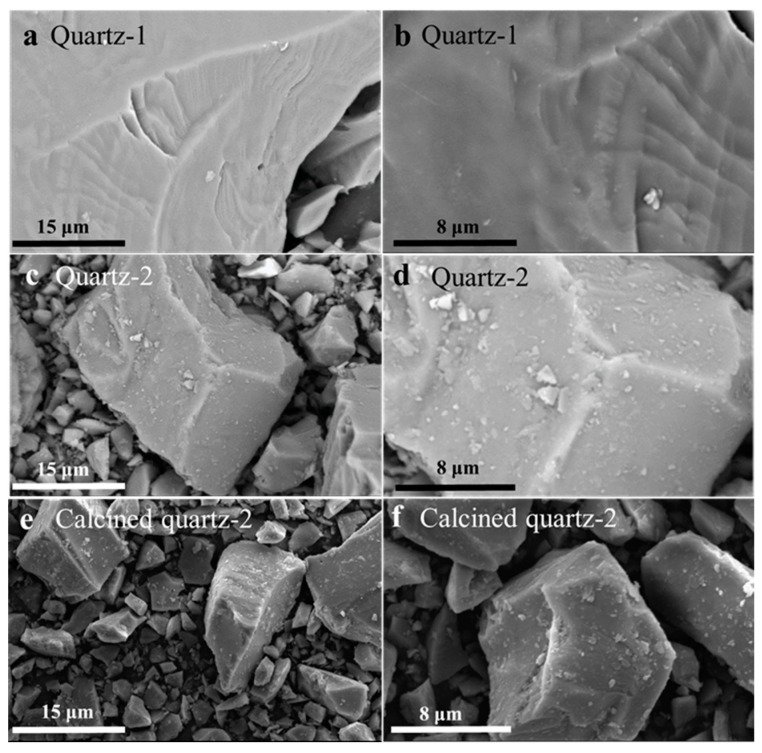
SEM images of (**a**,**b**) quartz-1, (**c**,**d**) quartz-2 and (**e**,**f**) calcined quartz-2.

Figure 5 shows the SEM images with different magnifications of quartz-1, quartz-2 and calcined quartz-2. The surface of quartz-1 shown in Figure 5a,b is clean. As shown in Figure 5c,d, many fine particles adhere to the surface of a coarse particle, resulting in a rough surface, which is caused by the adhesion of fine particles produced in the grinding process to the surface of coarse particles. Compared to quartz-2, the surface of calcined quartz-2 shown in Figure 5e,f has no obvious difference.

**Figure 6:**
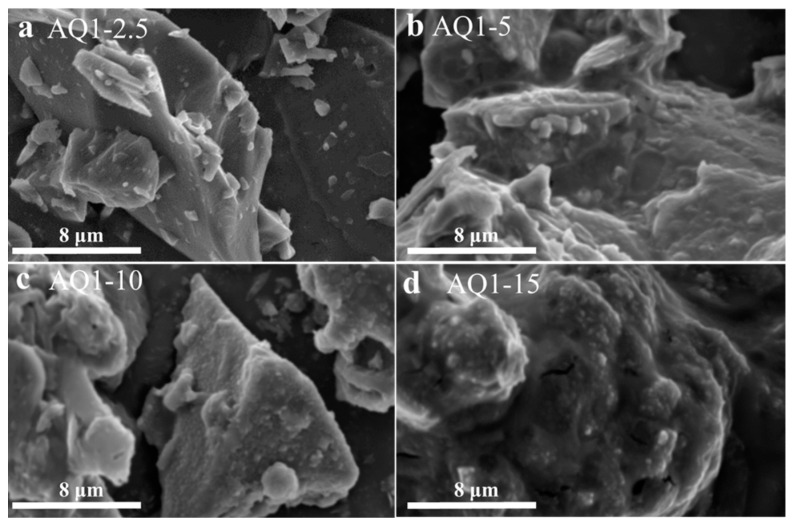
SEM images of quartz-1 activated with various content of NaOH: (**a**) AQ1-2.5, (**b**) AQ1-5, (**c**) AQ1-10 and (**d**) AQ1-15.

The morphologies of quartz-1 activated with various contents of NaOH are shown in Figure 6. As shown in Figure 6a, the quartz particles activated with 2.5% NaOH bonded to each other, which may be caused by the formed active materials on the particle surfaces. The quartz-1 activated with 5% NaOH (Figure 6b) exhibits rougher surfaces, and all the surfaces are covered with a layer of newly formed materials. The surfaces of AQ1-10 and AQ1-15 shown in Figure 6c,d are similar to that of AQ1-5. With the increase in alkali dosage, the cover of the quartz particles by the active material becomes more and more obvious. For AQ1-15 especially, the newly formed material wrapped around a large number of quartz particles to form a large block, indicating that the surfaces of all quartz particles were coated with active materials, and the amount of alkali for surface activation was enough.

**Figure 7:**
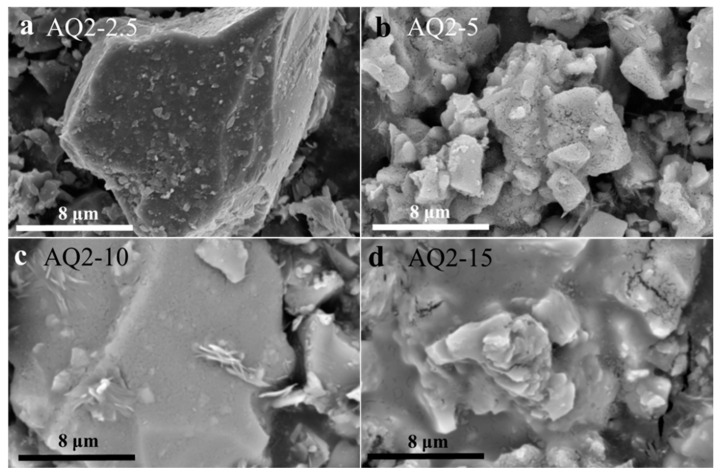
SEM images of quartz-2 activated with various contents of NaOH: (**a**) AQ2-2.5, (**b**) AQ2-5, (**c**) AQ2-10 and (**d**) AQ2-15.

Figure 7 shows the morphologies of quartz-2 activated with various content of NaOH. It can be observed that the morphologies of activated quartz-2 are similar to those of activated quartz-1. All the surfaces of quartz are covered with newly formed material at an alkali consumption of 5%, and a large block is observed at an alkali consumption of 15%. Hence, the SEM results show that 5% alkali dosage is sufficient to activate the surface of quartz-1 and quartz-2, which is much lower than the common used alkali dosage (10% or even 20% of the materials to be activated).

### 3.2. Activation Analysis

**Figure 8:**
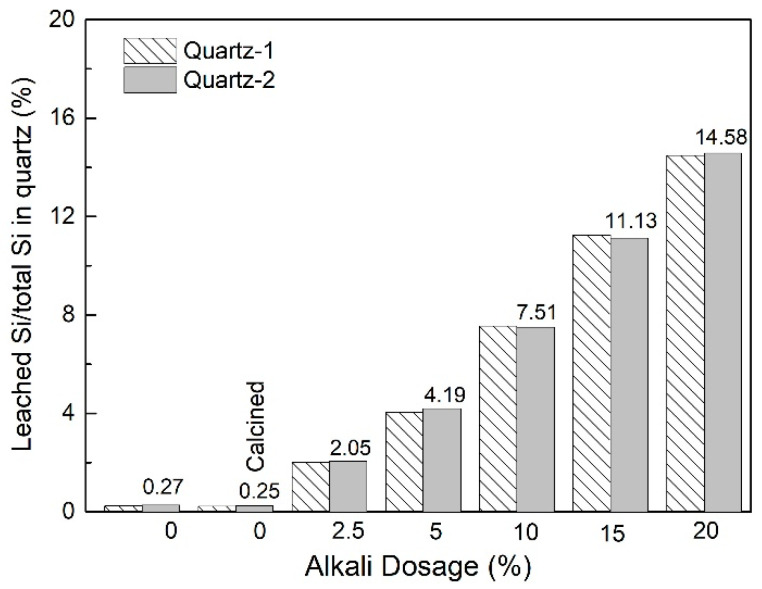
Leached Si content of quartz and activated quartz.

To evaluate the activation effect, the amount of the leached Si content is used to represent the amount of activated quartz. Figure 8 shows the leaching results of Si content in quartz and alkali-activated quartz. For quartz-1 and quartz-2, the percentages of leached Si to total Si in quartz and calcined quartz are about 0.27% and 0.25%, respectively, indicating that the reactivity of quartz in geopolymerization is low, and cannot be improved only by calcining at 550 °C for 60 min. When the alkali dosage used for activation was 2.5%, the percentage of leached Si to total Si in activated quartz-2 was 2.05% suggesting that part of the quartz is activated under the action of alkali and a high temperature. As the alkali dosage increases, the percentage of leached Si to total Si increases simultaneously. The leached Si to total Si in activated quartz-2 reached 14.58% at an alkali dosage of 20%. Since the surface Si to total Si of quartz is small, 14.58% of the quartz is activated suggesting that the inside of quartz was also activated at a high-alkali dosage. It can be observed that there is a liner relationship between the percentage of leached Si to total Si in activated quartz and the alkali dosage for activation. Therefore, both the surface and interior of the quartz particle can be effectively activated by alkali fusion at a high-alkali dosage. However, it is not an economic and reasonable way.

The leaching result of activated quartz-1 is consistent with that of activated quartz-2, and the percentage of leached Si to total Si is close to that of activated quartz-2 at the same alkali dosage. Therefore, the particle size of quartz has no obvious effect on the leaching of Si when the alkali fusion method is used to activate quartz. The leaching of Si is mainly determined by the alkali dosage. At an alkali dosage of 5%, the percentage of leached Si to total Si in activated quartz was 4.19%. The research on mechanical activation of quartz showed that the quartz surface can be effectively activated by a planetary mill at 1000 r/min for 60 min, and the percentage of leached Si to total Si was about 4.8% [28], which is close to the leaching result (4.19%) achieved at an alkali dosage of 5%. Therefore, the leaching result further confirms that the surface of quartz can be effectively activated at an alkali dosage of 5%.

### 3.3. Compressive Strength

**Figure 9:**
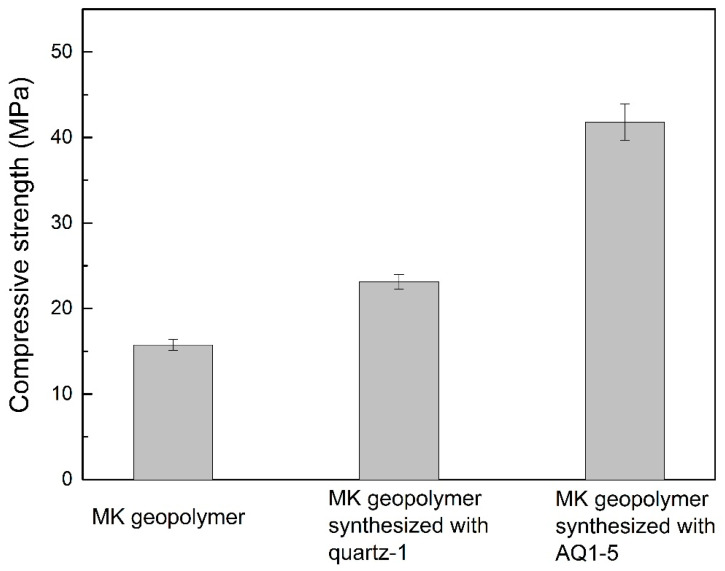
The compressive strength of the MK geopolymer and MK-based geopolymers synthesized with quartz-1 and AQ1-5.

**Figure 10:**
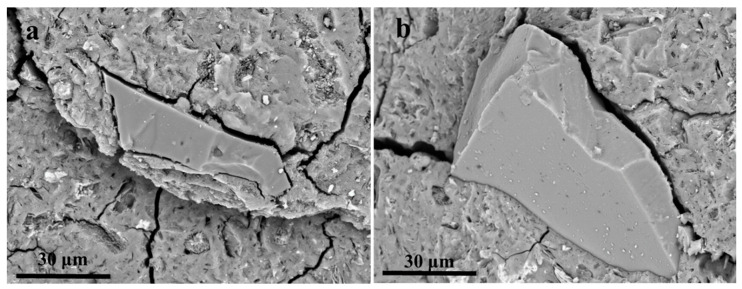
SEM images of the fracture surface of MK-based geopolymers synthesized with (**a**) quartz-1 and (**b**) AQ1-5.

Figure 9 presents the compressive strength of the MK geopolymer, MK-based geopolymer synthesized with quartz-1, and MK-based geopolymer synthesized with AQ1-5. The compressive strength of the MK geopolymer and MK-based geopolymer synthesized with quartz-1 are 15 MPa and 23 MPa, respectively, suggesting the reinforcement of quartz-1 as a filler material on MK geopolymer. The MK-based geopolymer synthesized with activated quartz-1 (AQ1-5) has a high compressive strength of 41 MPa. Therefore, compared to that of the MK-based geopolymer synthesized with quartz-1, the compressive strength of the MK geopolymer synthesized with AQ1-5 is significantly increased, indicating that the alkali fusion of quartz with an alkali dosage of 5% has a significant strengthening effect on the mechanical strength of the MK-based geopolymer. Figure 10 exhibits the SEM images of the fracture surface of geopolymers synthesized with quartz-1 and AQ1-5, respectively. As shown in Figure 10a, after the compression test, the quartz particle and geopolymer gel were completely separated, and a clear crack around the quartz can be observed. The complete separation of the quartz and geopolymer gel indicates that the interfacial interaction between the quartz and the gel is weak. However, after the compression test of the MK-based geopolymer synthesized with AQ1-5 shown in Figure 10b, the quartz particle and geopolymer gel are not completely separated. There is not only a large crack between the activated quartz and the geopolymer gel, but also a close combination. The close combination between the activated quartz and geopolymer gel demonstrates that the activated quartz surface enhances the interfacial interaction between the quartz surface and geopolymer gel, which may be the main reason for the high compressive strength of the MK geopolymer synthesized with AQ1-5 [28]. Therefore, the surface of quartz can be effectively activated at an alkali dosage of 5%, and can effectively enhance the strength of a geopolymer, which indicates that to prepare geopolymers from low-alkali-activated granite sawdust is a potential method.

## 4. Conclusions

It is feasible to effectively activate quartz fines through an alkali fusion process under low alkali conditions, and to achieve an enhanced compressive strength of metakaolin-based geopolymer synthesized with low-alkali-activated quartz. The main conclusions are as follows:

(1) The surface of a quartz particle can be effectively activated by an alkali fusion process at an alkali dosage of 5%. When the alkali dosage is more than 5%, the activated quartz particles will be agglomerated by the formed active materials. The higher the alkali dosage used for activation, the larger the agglomeration is.

(2) The amount of activated quartz is mainly determined by the amount of alkali used for activation. The leaching content of Si is positively correlated with the alkali dosage used for activation.

(3) The metakaolin-based geopolymer synthesized with quartz activated by an alkali dosage of 5% shows a high compressive strength of 41 MPa, which can be attributed to the enhanced interfacial interaction between the quartz and geopolymer gel.

This work suggests that low-alkali activation is a potential way to improve the geopolymerization ability of granite sawdust. In future work, it is worth exploring the geopolymerization ability of granite sawdust activated by low alkali consumption.

## Figures and Tables

**Table 1:** Chemical composition of MK.

Constituent	SiO_2_	Al_2_O_3_	Fe_2_O_3_	MgO	K_2_O	TiO_2_	CaO	V_2_O_3_	LOI
Content (Wt. %)	48.11	43.90	2.99	0.16	0.37	2.07	0.48	0.49	1.43

## Data Availability

Not applicable.
